# Supplementary material for: Characterization of Organellar-Specific ABA Responses during Environmental Stresses in Tobacco Cells and Arabidopsis Plants
Source: Cells. 2022 Jun 27;11(13):2039. doi: 10.3390/cells11132039 (PMC9265483; doi:10.3390/cells11132039)
Supplement: Supplementary file 1 [file cells-11-02039-s001.zip › cells-1773189-supplementary.pdf]

Supplementary Materials

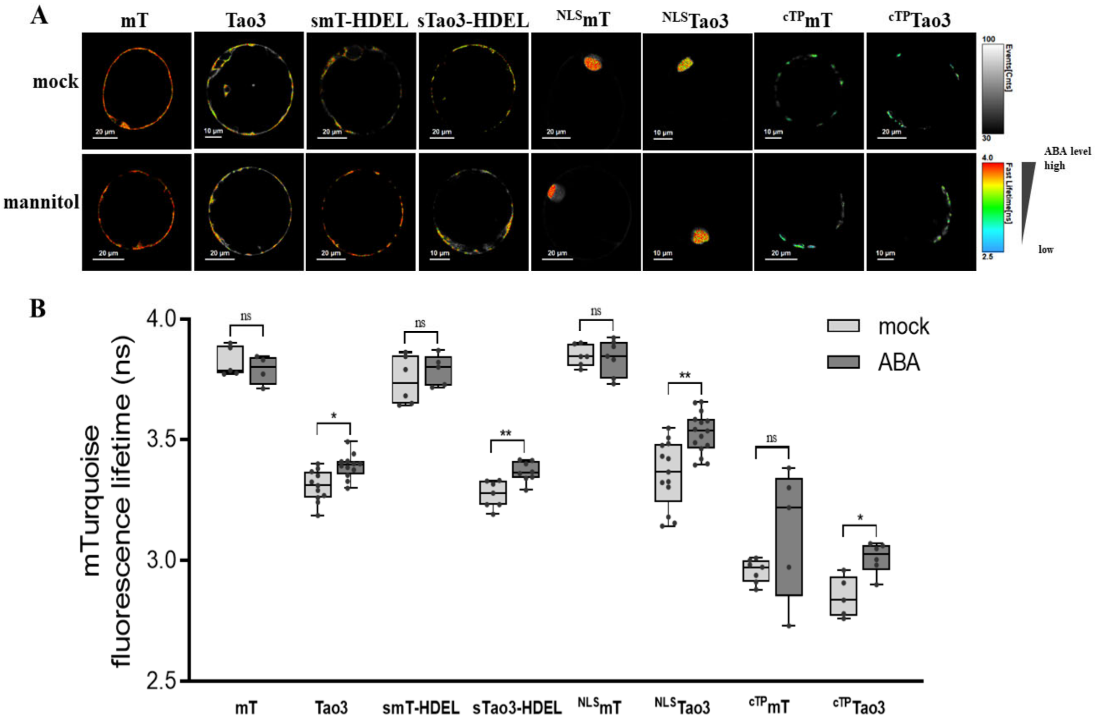

Figure S1. Organellar targeted Tao3s are sensitive to ABA treatment in tobacco protoplasts. (A,B) Representative FLIM images (**A**) and data (**B**) show increases in the τmT of organellar Tao3s, but not in that of donor-only (mTs) transfected cells upon exogenous ABA treatment (1 μM). FLIM data are presented as box plots showing all data points. Significance was calculated using Student's t test (* $p < 0.05$; ** $p < 0.01$; ns, not significant).

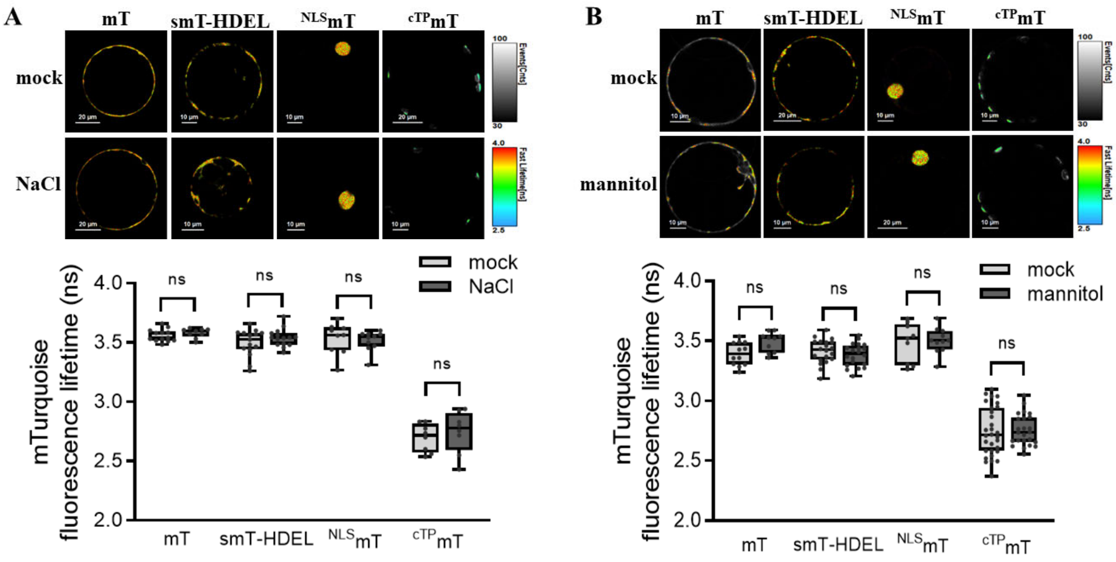

Figure S2. FLIM analysis reveals little changes in the fluorescence lifetime of the organellar targeted donor-only (mTurquoise) upon salt and osmotic stresses. (**A,B**) Representative FLIM images and data showing the insensitivity of the donor-only transfected cells to 10 mM NaCl (**A**), 50 mM mannitol (**B**) treatment. FLIM data are presented as box plots with all data points. Significance was calculated using Student's *t* test (ns, not significant).

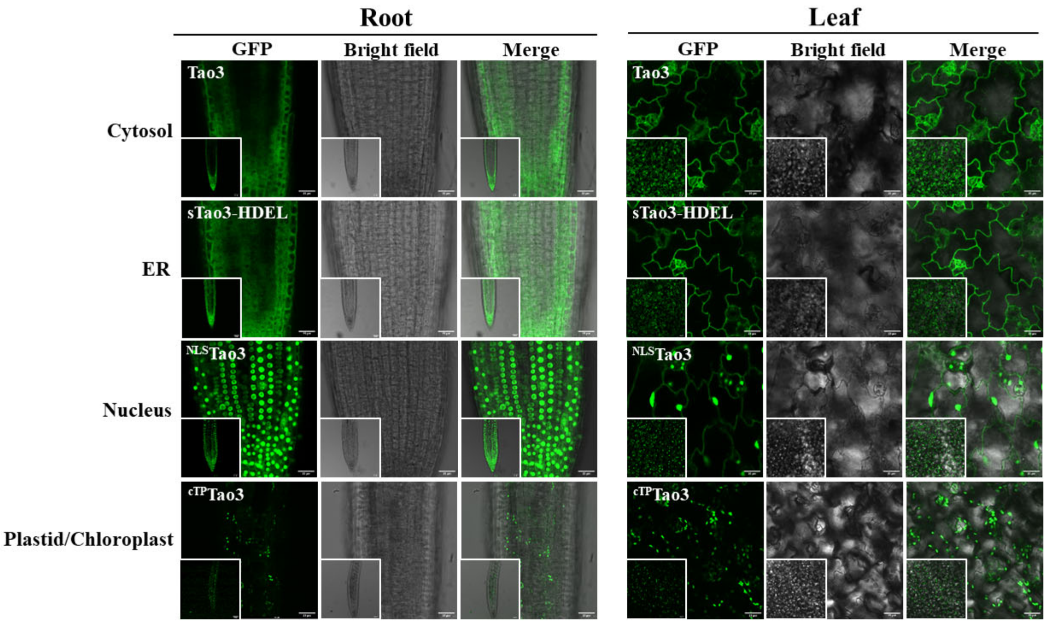

Figure S3. Targeting of Tao3s to different organelles in Arabidopsis plants. Confocal microscopic images of cytosolic, ER, nucleus and chloroplast/plastid Tao3s expressed in Arabidopsis roots (left) and leaves (right). Scale bar, 20 μm; insets, 50 μm.

Table S1. Oligonucleotides and Plasmids used in this study.

New fragment	Primers	Sequence (5'-3')	Template	Recipient Vector
ABAleon2.1_Tao3 (pYLZ23)	EcoRI-35S-F	TGCAGCgaattcctacgcagcaggtctcatcaagacg	pSF074*	pYLZ24 cut EcoRI/NheI
	NheI-35S-R	GCGgctagcatatagagagagagatagatttatagagag		
sABAleon2.1_Tao3-HDEL (pYLZ36)	linker3-ABI1-BamHI-F	GCTGATggatccggtggaggcgttgatcctgataatgaagcatacgaaatgccttctgaagaaggctatcaagattatgaaccggaggctggtggaggcagtgtgcctttgtatggttttac	ABAleon(Bar2.1)#	pYLZ21 cut BamHI
	BamHI-cpV-HDEL-R	CAGCggatccctaaagctcatcatgctcgatgttgtggcgga		
NLS-ABAleon2.1_Tao3 (pYLZ22)	EcoRI-35S-F	TGCAGCgaattcctacgcagcaggtctcatcaagacg	pSF074*	pYLZ23 cut EcoRI/NheI
	NheI-SV40-NLS-35S-R	GCGgctagcgacctttctcttcttctttggcatatatagagagagagatag		
cTP-ABAleon2.1_Tao3 (pYLZ28)	NheI-Bam4-F	GATCgctagcatgacggagactggagtaat	cTP-YC3.6 Camelon$	pYLZ23 cut NheI
	NheI-Bam4-R	AGCTgctagcggtggcggccgcaagcttacgcaa		
mT-PYR1-linker3 (pYLZ82)	EcoRI-35S-F	TGCAGCgaattcctacgcagcaggtctcatcaagacg	pSF074	pYLZ81 cut EcoRI/NheI
	NheI-35S-R	GCGgctagcatatagagagagagatagatttatagagag		
smT-PYR1-linker3-HDEL (pYLZ81H)	psp-PYR1-sosoo-F	gacgagctgtacaaggggcccatgccttcggagttaacaccagaagaac	pYLZ24	pYLZ21 cut psp/BamHI
	BanHI-HDEL-linker-PYR1-sosoo-R	tgaacgatctgcttcggatccctaaagctcatcatggcctccaccagcctccgg		
NLS-mT-PYR1-linker3 (pYLZ84)	EcoRI-35S-F	TGCAGCgaattcctacgcagcaggtctcatcaagacg	pYLZ22	pYLZ82 cut EcoRI/NheI
	NheI-SV40-NLS-35S-R	GCGgctagcgacctttctcttcttctttggcatatatagagagagagatag		
cTP-mT-PYR1-linker3 (pYLZ83)	NheI-Bam4-F	GATCgctagcatgacggagactggagtaat	cTP-YC3.6 Camelon	pYLZ82 cut NheI
	NheI-Bam4-R	AGCTgctagcggtggcggccgcaagcttacgcaa		

*, plasmids kindly provided by Dr. Peter Pimpl.

#, ABAleons, kindly provided by Dr. Waadt.

$, cTP-YC3.6 Camelon, kindly provided by Dr. Alex Costa.
